# Should we include dementia diagnosis or cognitive impairment to predict home care cost? An observational study using real-world data

**DOI:** 10.1186/s12913-025-13868-2

**Published:** 2025-12-22

**Authors:** Flurina Meier-Schwarzer, Nicole Probst-Hensch, Marc Höglinger, Matthias Schwenkglenks, Simon Wieser

**Affiliations:** 1 Winterthur Institute of Health Economics, School of Management and Law, Zurich University of Applied Sciences, Winterthur, Switzerland; 2 Department of Epidemiology and Public Health, Swiss Tropical and Public Health Institute, Allschwil, Switzerland; 3 University of Basel, Basel, Switzerland; 4 Health Economics Facility, Department of Public Health, University of Basel, Basel, Switzerland

**Keywords:** Dementia, Cognitive impairment, Home nursing care, Cost prediction, Tariffs

## Abstract

**Background:**

The reintroduction of dementia diagnosis into Medicare Advantage risk-adjustment models suggests that the condition independently influences Medicare costs. Similarly, Switzerland is considering specific tariffs for dementia patients in long-term care. However, the question of whether including dementia diagnosis meaningfully improves predictions of home nursing care costs is still under debate. Therefore, we examined whether including dementia diagnosis and/or symptoms of cognitive impairment, in addition to standard predictors, meaningfully improves the performance of home care cost predictions.

**Methods:**

Data from an observational multicentre study of eight Swiss home care providers across all language regions was analysed. The study included all patients undergoing the routine interRAI-Home Care Switzerland assessment of patient characteristics. We used administrative data from months two and three post-assessment including home nursing care costs covered by health insurers as well as generalised linear models with a gamma log-link function to predict costs. We assessed whether including a dementia diagnosis and/or a measure of cognitive impairment (cognitive performance scale, CPS) improved prediction in comparison to models with only standard predictors such as impairments in activities of daily living (ADL, e.g. bathing, dressing), instrumental activities of daily living (IADL, e.g. cooking, managing finances), and living situation (living alone).

**Results:**

Among the 1,035 patients in our dataset, 176 (17%) had a dementia diagnosis. Mean monthly home care costs for dementia patients were CHF 362 (Swiss francs) higher (mean: CHF 1,082; median: CHF 812) than for non-dementia patients (mean: CHF 720; median: CHF: 457). ADL and IADL were the most important predictors of home care costs. Introducing dementia diagnosis or cognitive impairment or both alongside ADL, IADL, living alone, age and gender did not materially change prediction performance (AIC remains unchanged, BIC changes + 1.09 to + 6.36 in the main model).

**Conclusion:**

Our findings give no support for the implementation of differentiated reimbursement tariffs or risk adjustment models for dementia patients or patients with cognitive impairment. More general predictors, such as ADL and IADL impairments seem to predict home nursing care costs adequately enough. However, further research with larger and more representative samples in other countries is necessary to validate these findings.

**Clinical trial number:**

Not applicable.

**Supplementary Information:**

The online version contains supplementary material available at 10.1186/s12913-025-13868-2.

## Background

The number of individuals living with dementia is expected to rise due to population aging [1, 2]. Most dementia patients are residing in home care settings due to a general shift from nursing homes toward home-based care [3–5]. As a result, the home care setting has become increasingly important in dementia care [1].

Dementia is a chronic, progressive condition, and individuals with dementia have evolving care needs depending on the stage of the disease and associated symptoms [1, 6, 7]. Early in the course of the disease, patients often require supervision or assistance with instrumental activities of daily living (IADLs), such as household chores, shopping, or managing finances [8, 9]. Individuals with IADL impairments may require guardianship, support with household tasks, or accompaniment to medical appointments – services frequently provided by informal caregivers [10, 11]. As the disease progresses, impairments in basic activities of daily living (ADLs) – such as bathing and dressing – become more prominent and more often necessitate formal care [10, 11].

The costs of care for dementia patients living at home reflect this trajectory. Informal care accounts for the majority of societal costs of home care, with estimates ranging from 60% to 84% of total costs [5]. Home aid services account for the second largest part of societal costs [12], while formal nursing care accounts for the smallest part [12].

Despite accounting for only a small share of total societal home care costs, formal home nursing care has high importance from a health care payer perspective. It is typically reimbursed through health insurance systems, whereas informal care does not usually add to costs borne by insurers [5]. Additionally, home aid services are not, or only partially, covered by some health care payers, such as Medicaid in the United States or Switzerland’s mandatory health insurance [13, 14].

Although home nursing care represents a cost burden for insurers, it may also help delay or prevent the institutionalisation of dementia patients [3]. Since formal care costs are much higher in institutional settings than in home care [5, 15–17], maintaining a robust formal home care infrastructure is crucial - and requires appropriate reimbursement.

As with other conditions that generate formal home care costs, the primary cost driver in dementia care is ADL impairment [11, 12]. Whether dementia itself, or the degree of cognitive impairment, independently drives home nursing care costs remains debated [11, 16, 18–21]. This uncertainty contributed to the controversy surrounding the 2020 reintroduction of dementia as a factor in Medicare Advantage risk-adjustment models for non-institutionalised patients [22]. This discussion is very topical in Switzerland as new long-term care tariffs need to be developed over the next seven years [23]. At the same time, policymakers are considering whether to implement differentiated tariffs for individuals with and without dementia for home nursing care.

Therefore, we investigated whether including either all-cause dementia diagnosis or cognitive impairment – alongside standard predictors such as ADL and IADL impairments – enhances the performance of home nursing care cost predictions. 

## Methods

### Study population and data sources

We analysed secondary data from a longitudinal observational multicentre study. Data was collected from eight non-profit home care service providers operating across all three Swiss language regions (German, French, and Italian). The study included all home care patients who underwent a routine assessment or reassessment using the standardised interRAI-Home Care Switzerland (interRAI) questionnaire between May and November 2022. The original dataset comprised 1,079 patients.

Our analyses were based on the following two data sources: (1) administrative records detailing the number of minutes of home nursing care provided as the basis for accrued costs (2) interRAI assessment records routinely recorded by case-leading home care nurses as a basis for care planning and negotiations with health insurers. The interRAI captures a wide range of information, including patients’ health status, treatments, functional abilities, behaviour, living situation, involvement of informal caregivers, and prior healthcare utilisation. It also includes physician-diagnosed dementia, as well as the variables used to calculate the Cognitive Performance Scale (CPS) [24] – a measure validated against the Mini-Mental State Examination and shown to be highly valid for assessing cognitive impairment such as in people with dementia [25]. The InterRAI is typically conducted every six months and earlier if a patient’s condition changes substantially.

### Outcome

To calculate the outcome variable – average home nursing care costs covered by health insurers – we used administrative data detailing the number of minutes of home nursing care by tariff group. Each patient’s average health insurance costs in the second and third month after study inclusion were calculated and averaged over both months. We only used data from the second and third months following the interRAI assessment, as costs from the first month may substantially be influenced by care planning itself. As a result, 44 patients were excluded from analysis: 30 received care only in the first month, 5 entered a nursing home, 2 were hospitalised, and 7 died within the first month. The final analytical sample included 1,035 patients.

Costs were calculated based on the current tariff system for home nursing care in Switzerland. It defines three time tariffs paid by health insurance: (1) basic nursing care: e.g. assistance with personal hygiene: 54.60 (CHF; 1 CHF ≈ 1 USD ≈ 1 EUR in 2022) per hour, (2) specialised nursing care, e.g. measurement of vital signs or preparation and administration of medication: CHF 63.00 per hour, and (3) care planning, counselling and coordination with other health care providers: CHF 79.80 per hour (Art. 7a, OBMHI [26]).

Funding of home nursing care is based on these three time tariffs and an individual care plan for each patient. It defines the number of care minutes per tariff group. Care planning is based on nurses’ assessment of patient situations, taking into account informal care delivered by caring relatives. In principle, nurses face no time restraints in their care planning, the only exception being that the care plan must be accepted and prescribed by a general practitioner and insurers can review the case if it surpasses 60 h of home care per quarter (Art. 8c OBMHI [26]). Furthermore, contributions on the highest tariff level are restricted to care situations that are complex or instable, except for the time for care planning itself (Art. 7 OBMHI [26]).

The mandatory health insurance in Switzerland only funds home nursing care and excludes all kinds of support services such as household help, meals on wheels, help with cooking or managing finances, social support or accompaniment, e.g. to doctor’s appointments. Our analyses are restricted to this Swiss definition of home nursing care.

All cost values were standardised to a 30-day month.

### Predictors

The basic model included interRAI-derived indicators of impairments in ADLs and IADLs, living situation (alone/cohabiting), as well as age (years) and gender (female/male). It is based on previous research on predictors of home care costs in dementia patients [11, 15, 16, 18].

The ADL score ranges from 0 to 10, reflecting the number of activities in which the patient requires any assistance [27]. The considered ADL impairments were bathing/showering, personal hygiene, dressing/undressing the upper body, dressing/undressing the lower body, walking indoors on the same floor, getting around the house, toilet transfer, toilet use, bed mobility, and eating/drinking.

The IADL score ranges from 0 to 8, again reflecting the number of activities in which the patient requires any assistance. Considered impairments were difficulties with meal preparation, household chores, money management, medication management, telephone use, stair use, shopping, and public transport use. All-cause dementia diagnosis was based on reports by home care nurses about the presence of a written physician-diagnosed Alzheimer’s disease or any other form of dementia. This information was extracted directly from the InterRAI.

As underdiagnosis of dementia is widespread worldwide, as well as in Switzerland [28, 29], we used the Cognitive Performance Scale (CPS) to capture people that show symptoms of cognitive impairment without having a formal dementia diagnosis. The CPS showed good validity against the mini mental state examination in the nursing home and in the home care setting [25, 30]. The Cognitive Performance Scale (CPS) that categorises cognitive function into seven levels: (0) intact, (1) borderline intact, (2) mild, (3) moderate, (4) moderate/severe, (5) severe, and (6) very severe. Any patient that showed a CPS of 2 or higher [25]was labelled as having a cognitive impairment.

### Statistical analyses

Home care costs were estimated using generalised linear models (GLMs) with a gamma distribution and log link function to account for the skewed cost distribution. The basic model included age, gender, ADL impairments, IADL impairments, and living situation. To assess the predictive value of dementia-related variables, we tested three extended models: (1) including dementia diagnosis alone, (2) including cognitive impairment alone, and (3) including both dementia diagnosis and cognitive impairment.

We performed six additional analyses to see whether they improved the previously-described prediction models. Firstly, we used different specifications of the cognitive impairment variable by using (a) a cut-off of CPS ≥ 1 instead of CPS ≥ 2 and (b) by adding it as a variable with four categories (0: intact, 1: borderline intact, 2: mild, 3: moderate, moderate/severe, severe and very severe) instead of a variable with two categories (0: intact or borderline intact or 1: mild, moderate, moderate/severe, severe and very severe). Secondly, we added age as a quadratic term to the models. Thirdly, we added interaction terms between ADL impairments and either dementia diagnosis, cognitive impairment or both to the above-mentioned models. Fourthly, we added an interaction term between gender and either dementia diagnosis, cognitive impairment or both to the models. Lastly, we tested the addition of several new control variables as predictors to the basic model. These additional predictors were: an indicator for the home care service providers, somatic comorbidities and restrictions in mobility. The first was added to account for local practice patterns and unmeasured regional factors. The second was a count of the following conditions: coronary heart disease, cardiac insufficiency, cerebrovascular insult (CVI), chronic obstructive pulmonary disease, cancer, diabetes, hemi-, para- or tetraplegia, Parkinson’s disease, multiple sclerosis, fracture/hip fracture (in the past 30 days), pneumonia or urinary infection (in the past 30 days). The third predictor was a variable for mobility restrictions that could take the following forms: (0) mobile without aids, (1) mobile with aids (e.g. cane, crutch, walking aid, pushes wheelchair), (2) mobile with wheelchair, (3) bedridden.

We also conducted two subgroup analyses. In the first one, we examined whether cognitive impairment could predict costs among patients without a dementia diagnosis – potentially identifying individuals with undiagnosed cognitive decline. In the second, we assessed whether cognitive impairment influenced costs among patients with a dementia diagnosis.

We used the Bayesian Information Criterion (BIC) and Akaike Information Criterion (AIC) to evaluate prediction performance in all prediction models [31]. The BIC and AIC are model selection criteria used to compare statistical models by balancing model fit and complexity, with AIC emphasizing predictive accuracy and BIC imposing a stronger penalty for model complexity to favour more parsimonious models. Lower BIC or AIC values indicate a better model.

Due to very low missing rates (see Table 1), no imputation strategy was applied. All analyses were conducted using Stata version 18.0.

### Ethical approval

Our study adheres to the declaration of Helsinki. The Ethics Commission of Northern and Central Switzerland concluded that the study our analyses were based on does not fall within the scope of the Human Research Act (BASEC-Nr. Req-2022-00286) and does not require ethical approval or the formal informed consent of home nursing care patients involved. The data analysed in this study is not publicly available due to data protection laws under which participating home care service providers operate (Art. 5c, digit 2, FADP [32]).

## Results

Of 1,035 home care patients included in the study, 176 (17%) had a physician-diagnosed all-cause dementia (Table 1). The overall age range of our participants reached from 25 to 105 years with a mean age of 80.2 years and a median age of 83 years. Patients with dementia diagnosis were on average 4.1 years older and more often had at least one ADL dependency compared to those without (77% versus 66%). Differences in having any IADL impairment (98% vs. 95%) and in the prevalence of living alone (57% vs. 60%) were modest. However, a higher proportion of patients with dementia had multiple IADL impairments. Additionally, cognitive impairment showed a pronounced disparity with 76% of patients with dementia exhibiting a CPS of 2 or more, compared to only 20% of those without such a diagnosis.

**Table 1:** Descriptive characteristics of patients by all-cause dementia diagnosis

	patients with dementia* diagnosis (*N* = 176, 17%)		patients without dementia* diagnosis (*N* = 859, 83%)	
Characteristic	mean, *N*	SD, %	mean, *N*	SD, %
*Home care use (min/month)*	1137	1032	743	823
*Age (years)*	83.6	7.9	79.5	12.2
*Gender*				
women	125	71%	512	60%
men	51	29%	347	40%
*ADL impairment score*				
none	40	23%	296	34%
1	28	16%	192	22%
2	20	11%	93	11%
3	15	9%	69	8%
4	16	9%	64	7%
5	14	8%	34	4%
6	8	5%	31	4%
7	4	2%	17	2%
8	6	3%	22	3%
9	13	7%	23	3%
10	12	7%	18	2%
*IADL impairment score*				
none	3	2%	44	5%
1	6	3%	75	9%
2	13	7%	126	15%
3	19	11%	143	17%
4	25	14%	147	17%
5	38	22%	160	19%
6	32	18%	97	11%
7	26	15%	52	6%
8	13	7%	15	2%
*Living situation*				
living alone	101	57%	516	60%
not living alone	75	42%	341	40%
*Cognitive functioning*				
Intact	13	7%	488	57%
Borderline intact	29	16%	203	24%
Mild	80	45%	138	16%
Moderate	23	13%	18	2%
Moderate/Severe	0	0%	2	0%
Severe	28	16%	9	1%
Very severe	3	2%	1	0%

ADL: activities of daily living, IADL: instrumental activities of daily living; min: minutes; N: number; SD: standard deviation; *all cause dementia includes Alzheimer’s disease and any other form of dementia

Compared to the total Swiss population receiving home care, our sample was skewed toward older individuals. Specifically, the oldest age group (80 years and older) was overrepresented with 61%, compared to only 39% in the national home care population [33]. In contrast, the youngest age group (20–64 years) was underrepresented with 11% compared to 32% of the national home care population [33]. The 65–79 age group was proportionally represented, comprising 28% of our sample versus 29% in the national population [33].

**Fig. 1:**
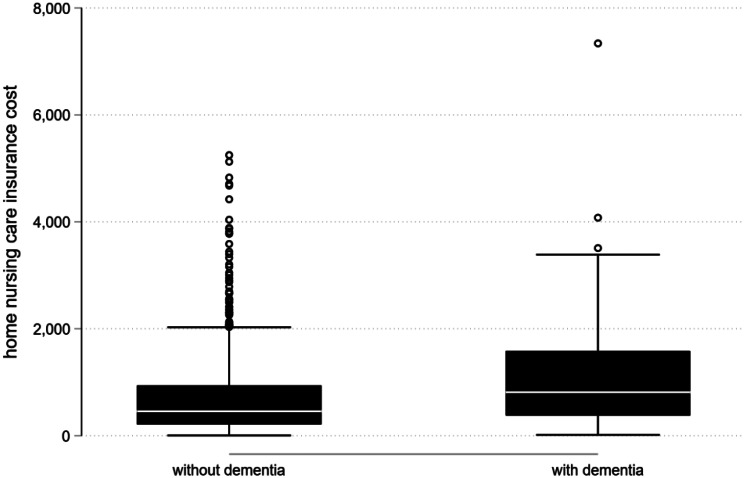
Home nursing care costs by dementia diagnosis

Costs for home nursing care were markedly higher in individuals with dementia diagnosis compared to those without (Fig. 1). The average monthly cost for dementia patients was CHF 1,082, with a median of CHF 812. In contrast, clients without a dementia diagnosis incurred mean costs of CHF 720 and a median of CHF 457.

### Univariable prediction

In order to estimate the predictive value of each predictor independently of all other predictors as a baseline value, we performed univariable predictions for all predictors used in the subsequent multivariate models. Univariate analyses identified ADL impairment as the strongest individual predictor of home care costs (BIC: -6,291.71, AIC: 15.14; Table 2), followed by IADL impairment (BIC: -6,188.45, AIC: 15.23), cognitive impairment (BIC: -6,134.02, AIC: 15.29), and dementia diagnosis (BIC: -6,124.87, AIC: 15.30). Other variables demonstrated more limited predictive value.

**Table 2:** Coefficients of monthly home care costs predictors and model BIC based on univariable GLM models

			CI			
	Coeff	*p*	Lower	Upper	BIC	AIC
age	0.004	0.102	-0.001	-0.010	-6101.23	15.32
male gender	0.050	0.442	-0.080	0.180	-6098.95	15.32
ADL impairment score	0.155	< 0.001	0.134	0.177	-6291.71	15.14
IADL impairment score	0.159	< 0.001	0.127	0.191	-6188.46	15.23
living alone	-0.040	0.499	-0.170	0.080	-6091.16	15.33
dementia diagnosis	0.410	< 0.001	0.240	0.580	-6124.87	15.30
cognitive impairment	0.399	< 0.001	0.259	0.538	-6134.02	15.29

ADL: activities of daily living, AIC: Akaike Information Criterion, BIC: Bayesian Information Criterion, CI: Confidence Interval, IADL: instrumental activities of daily living

### Multivariable prediction models

**Table 3:** Coefficients of monthly home care costs predictors and model BIC based on multivariable GLM models

	Basic Model				Model 1				Model 2				Model 3			
*BIC*	***-6281.88***				***-6280.97***				***-6279.73***				***-6275.52***			
*AIC*	***15.11***				***15.11***				***15.11***				***15.11***			
		**CI**				**CI**				**CI**				**CI**		
	**coeff**	**lower**	**upper**	**P**	**coeff**	**lower**	**upper**	**p**	**coeff**	**lower**	**upper**	**p**	**coeff**	**lower**	**upper**	**P**
age	0.003	-0.002	0.008	0.239	0.003	-0.002	0.008	0.315	0.003	-0.002	0.008	0.300	0.002	-0.003	0.008	0.336
male gender	0.075	-0.043	0.192	0.215	0.090	-0.028	0.209	0.135	0.080	-0.037	0.198	0.181	0.090	-0.029	0.208	0.138
ADL impairment	0.143	0.119	0.167	< 0.001	0.142	0.118	0.166	< 0.001	0.141	0.117	0.165	< 0.001	0.141	0.116	0.165	< 0.001
IADL impairment	0.076	0.042	0.110	< 0.001	0.069	0.034	0.103	< 0.001	0.067	0.032	0.102	< 0.001	0.065	0.030	0.100	< 0.001
living alone	0.278	0.158	0.397	< 0.001	0.273	0.153	0.392	< 0.001	0.279	0.160	0.398	< 0.001	0.275	0.155	0.394	< 0.001
dementia diagnosis					0.206	0.053	0.359	0.009					0.156	-0.015	0.326	0.073
cognitive impairment									0.156	0.028	0.283	0.017	0.097	-0.046	0.239	0.184

ADL: activities of daily living, AIC: Akaike Information Criterion, BIC: Bayesian Information Criterion, CI: Confidence Interval, IADL: instrumental activities of daily living

To evaluate the combined prediction performance of standard predictors on home care costs including ADL impairment, IADL impairment, living alone, age, and gender, we built the basic multivariable model (Table 3). It yielded a BIC of -6,281.88 and an AIC of 15.11. By adding dementia diagnosis and/or cognitive impairment to this basic model, we could evaluate if these additional variables improved model performance and if they show an independent predictive value on home care costs.

Adding dementia diagnosis or cognitive impairment slightly decreased prediction performance of the model based on the BIC (dementia diagnosis: -6280.97, cognitive impairment: -6279.73), whereas the AIC remained at the same level (15.11 for both models). Both predictors showed some predictive value (dementia diagnosis: *p* = 0.009, cognitive impairment: 0.017). Incorporating both dementia diagnosis and cognitive impairment resulted in a further reduced prediction performance based on the BIC (dementia diagnosis: -6275.52) but in a stable AIC (15.11) compared to all other models.

### Additional analyses

In order to test whether the previously estimated models could be improved by different categorisation of predictors or model specification, i.e. by adding interactions, we performed six additional analyses. Three additional analyses improved prediction performance: the introduction of an interaction between ADL impairments and cognitive impairment, the introduction of an interaction between ADL impairments and dementia diagnosis as well as cognitive impairment as well as the introduction of three additional prediction variables. The first model yielded a slightly decreased BIC (− 6283.35) and AIC (15.10) compared to in the basic model (BIC: -6281.88, AIC: 15.11; Table 4). The second model yielded only a slightly dec reased AIC (15.10) but also a strongly increased BIC (-6273.10; Tabel 4).

**Table 4:** Multivariable prediction of home care costs using a GLM model with a log-link and an interaction between ADL impairment and cognitive impairment and an interaction between ADL impairment and dementia diagnosis as well as cognitive impairment

	Basic Model				Model 2 (including interaction ADL impairment*cognitive impairment)				Model 4 (including interaction ADL impairment*dementia diagnosis and ADL impairment*cognitive impairment)			
*BIC*	***-6281.88***				***-6283.35***				***-6273.10***			
*AIC*	***15.11***				***15.10***				***15.10***			
		**CI**				**CI**				**CI**		
	**coeff**	**lower**	**upper**	**p**	**coeff**	**lower**	**upper**	**p**	**coeff**	**lower**	**upper**	**P**
age	0.003	-0.002	0.008	0.239	0.003	-0.002	0.008	0.305	0.002	-0.003	0.007	0.347
male gender	0.075	-0.043	0.192	0.215	0.082	-0.035	0.199	0.168	0.093	-0.025	0.211	0.123
ADL impairment	0.143	0.119	0.167	< 0.001	0.068	0.143	0.206	< 0.001	0.176	0.144	0.207	< 0.001
IADL impairment	0.076	0.042	0.110	< 0.001	0.262	0.033	0.103	< 0.001	0.065	0.030	0.100	< 0.001
living alone	0.278	0.158	0.397	< 0.001	0.174	0.143	0.381	< 0.001	0.256	0.137	0.376	< 0.001
dementia diagnosis									0.214	-0.030	0.457	0.085
ADL impairment*dementia diagnosis									-0.013	-0.072	0.046	0.670
cognitive impairment					0.379	0.199	0.559	< 0.001	0.304	0.100	0.508	0.003
interaction ADL impairment* cognitive impairment					-0.081	-0.125	-0.037	< 0.001	-0.077	-0.131	-0.024	0.004

ADL: activities of daily living, AIC: Akaike Information Criterion, BIC: Bayesian Information Criterion, CI: Confidence Interval, IADL: instrumental activities of daily living

The introduction of three additional prediction variables improved the basic model (BIC: -6304.82, AIC: 15.05; Table 5). With regard to the other models, this additional analysis did not generally alter our main findings: the addition of dementia diagnosis or cognitive impairment decreased model prediction accuracy slightly based on the BIC but remained the same based on the AIC (dementia diagnosis: BIC: -6303.52, AIC: 15.05; cognitive impairment: BIC: -6302.42, AIC: 15.05). The addition of both cognitive impairment and dementia diagnosis decreased prediction accuracy even further based on the BIC (BIC: -6298.11) but remained the same based on the AIC (AIC: 15.05).

**Table 5:** Multivariable prediction of home care costs using a GLM model with a log-link and additional predictors in the basic model

	Basic Model with additional variables				Model 2 with additional variables				Model 3 with additional variables				Model 4 with additional variables			
*BIC*	***-6304.82***				***-6303.52***				***-6302.42***				***-6298.11***			
*AIC*	***15.05***				***15.05***				***15.05***				***15.05***			
		**CI**				**CI**				**CI**				**CI**		
	**coeff**	**lower**	**upper**	**p**	**coeff**	**lower**	**upper**	**p**	**coeff**	**lower**	**upper**	**p**	**coeff**	**lower**	**upper**	**p**
age	0.003	-0.002	0.007	0.305	0.002	-0.003	0.007	0.383	0.002	-0.003	0.007	0.397	0.002	-0.003	0.007	0.425
male gender	0.054	-0.059	0.167	0.348	0.066	-0.048	0.179	0.256	0.057	-0.055	0.170	0.319	0.065	-0.049	0.178	0.263
ADL impairment	0.119	0.093	0.144	< 0.001	0.115	0.089	0.140	< 0.001	0.114	0.088	0.140	< 0.001	0.113	0.087	0.138	< 0.001
IADL impairment	0.075	0.042	0.107	< 0.001	0.068	0.035	0.101	< 0.001	0.067	0.034	0.100	< 0.001	0.064	0.031	0.098	< 0.001
living alone	0.233	0.117	0.349	< 0.001	0.230	0.114	0.346	< 0.001	0.238	0.122	0.353	< 0.001	0.234	0.118	0.349	< 0.001
home care provider																
2	-0.028	-0.348	0.291	0.863	-0.036	-0.356	0.284	0.825	0.011	-0.309	0.331	0.946	-0.009	-0.331	0.312	0.955
3	0.487	0.225	0.748	< 0.001	0.444	0.181	0.707	0.001	0.499	0.238	0.760	< 0.001	0.462	0.197	0.726	0.001
4	0.368	0.110	0.625	< 0.001	0.365	0.107	0.623	0.006	0.367	0.110	0.624	0.005	0.365	0.107	0.622	0.005
5	-0.113	-0.365	0.140	0.382	-0.137	-0.390	0.117	0.290	-0.109	-0.361	0.143	0.398	-0.128	-0.381	0.125	0.322
6	0.618	0.375	0.862	< 0.001	0.597	0.354	0.841	< 0.001	0.611	0.369	0.854	< 0.001	0.598	0.355	0.842	< 0.001
7	0.487	0.220	0.753	< 0.001	0.460	0.192	0.727	0.001	0.477	0.211	0.743	< 0.001	0.460	0.193	0.727	0.001
8	0.592	0.348	0.836	< 0.001	0.534	0.287	0.782	< 0.001	0.570	0.326	0.814	< 0.001	0.535	0.288	0.782	< 0.001
comorbidity	0.045	-0.005	0.095	0.079	0.054	0.004	0.105	0.035	0.048	-0.001	0.098	0.057	0.054	0.004	0.104	0.035
mobility	0.157	0.060	0.253	0.001	0.176	0.079	0.274	< 0.001	0.180	0.082	0.278	< 0.001	0.187	0.088	0.285	< 0.001
dementia diagnosis					0.209	0.058	0.360	0.007					0.158	-0.008	0.324	0.062
cognitive impairment									0.158	0.032	0.284	0.014	0.101	-0.038	0.239	0.154

ADL: activities of daily living, AIC: Akaike Information Criterion, BIC: Bayesian Information Criterion, CI: Confidence Interval, CPS: Cognitive Performance Scale, IADL: instrumental activities of daily living

All other additional analyses did not improve prediction accuracy. In all models the AIC remained at 15.11 as in the basic model. We, therefore, henceforth only discuss changes in BIC.

Changing the cut-off threshold for cognitive impairment from 2 (mild cognitive impairment) to 1 (borderline intact) improved the performance of Model 2 by decreasing BIC slightly from − 6281.88 to -6283.03 (see online supplement, Table A2). Both other models performed worse than the basic model as in the main analyses.

Adding cognitive impairment with four categories (0: intact, 1: borderline intact, 2: mild, 3: moderate, moderate/severe, severe and very severe) instead of two (0: intact or borderline intact, 1: mild, moderate, moderate/severe, severe and very severe) did not improve prediction performance. Specifically, Model 3 yielded a BIC of -6,269.62, and Model 4 a BIC of -6,265.21 (see online supplement, Table A3).

Adding a quadratic term for age into the prediction model did not improve model performance. The basic model showed a BIC of -6274.98 and age squared a very small coefficient − 0.0000275 (*p* = 0.824). All other models also showed lower BIC values. Specifically, Model 2 (with dementia diagnosis) yielded a BIC of -6,274.04, Model 3 (with cognitive impairment) a BIC of -6,272.80, and Model 4 (with both) a BIC of -6,268.59 (see online supplement, Table B).

Introducing interaction terms between ADL impairments and dementia diagnosis did not improve model performance. Specifically, Model 2 (interaction with dementia diagnosis) yielded a BIC of -6,278.77 (see online supplement, Table C1).

Introducing interaction terms between gender and either dementia diagnosis, cognitive impairment or both did not improve model performance either. Specifically, Model 2 (interaction with dementia diagnosis) yielded a BIC of -6,275.15, Model 3 (interaction with cognitive impairment) a BIC of -6,275.83, and Model 4 (interaction with dementia diagnosis and cognitive impairment) a BIC of -6,264.06 (see online supplement, Table D1)

### Subgroup analyses on the effect of cognitive impairment

In order to investigate if our cognitive impairment variable could improve home care cost prediction in a population (1) without dementia diagnosis or (2) with dementia diagnosis, we performed subgroup analyses in these two mutually exclusive subgroups (Table 6). Among patients without a dementia diagnosis, adding cognitive impairment to the model only marginally improved prediction performance based on the AIC (basic model: AIC: 15.90; model with cognitive impairment: AIC: 15.89) but resulted in poorer prediction accuracy based on the BIC (basic model: BIC: -760.37; model with cognitive impairment: BIC: -756.22). Among patients with dementia adding cognitive impairment to the model decreased prediction performance based on both the AIC and the BIC (basic model: AIC: 14.96; BIC: -5034.57; model with cognitive impairment: AIC: 14.96; BIC: -5028.60). In both subgroup analyses cognitive impairment failed to show predictive value (patients without a dementia diagnosis: *p* = 0.160; patients with a dementia diagnosis: *p* = 0.353).

**Table 6:** Associations of predictors and monthly home care costs based on adjusted GLM models in subgroups

	People without dementia diagnosis (*N* = 859)								People with dementia diagnosis (*N* = 176)							
	Basic model				Model 1				Basic model				Model 1			
*BIC*	***-760.37***				***-756.22***				***-5034.57***				***-5028.6***			
*AIC*	***15.9***				***15.89***				***14.96***				***14.96***			
		**CI**				**CI**				**CI**				**CI**		
	**coeff**	**lower**	**upper**	**p**	**Coeff**	**lower**	**upper**	**p**	**coeff**	**lower**	**upper**	**P**	**coeff**	**lower**	**upper**	**p**
age	-0.007	-0.020	0.007	0.334	-0.007	-0.021	0.006	0.294	0.004	-0.002	0.009	0.198	0.004	-0.002	0.009	0.208
male gender	-0.049	-0.298	0.200	0.698	-0.024	-0.279	0.232	0.856	0.111	-0.021	0.243	0.099	0.109	-0.023	0.241	0.106
ADL impairment	0.088	0.044	0.131	< 0.001	0.084	0.039	0.129	< 0.001	0.156	0.128	0.185	< 0.001	0.156	0.127	0.184	< 0.001
IADL impairment	0.100	0.029	0.171	0.006	0.090	0.017	0.164	0.016	0.065	0.026	0.104	0.001	0.063	0.024	0.102	0.002
living alone	0.287	0.032	0.543	0.027	0.292	0.033	0.551	0.027	0.257	0.123	0.391	< 0.001	0.259	0.124	0.393	< 0.001
cognitive impairment					0.195	-0.077	0.468	0.160					0.077	-0.086	0.240	0.353

ADL: activities of daily living, AIC: Akaike Information Criterion, BIC: Bayesian Information Criterion, CI: Confidence Interval, IADL: instrumental activities of daily living

## Discussion

Our analysis shows that the average costs of formal home nursing care are higher for patients with dementia diagnosis than for those without. Hence, individuals with dementia incur greater expenses not only for informal care and home support services [5, 11, 12], but also for home nursing care. This somewhat contradicts a previous study finding only small differences in Medicare costs between home care patients with and without dementia in the US ^19^.

The best predictors of higher home nursing care costs in dementia patients or those with cognitive impairment are limitations in ADL and IADL. Adding dementia diagnosis or cognitive impairment to the prediction model did not improve prediction performance. These findings remained generally the same in several additional analyses. Hence, we found no support for the implementation of differentiated reimbursement tariffs or risk-adjustment models for dementia patients. However, our sample size was small and included patients from only eight home care service providers. Therefore, further research with a larger and more representative data sample is necessary to validate and extend our findings. When Switzerland introduces new tariffs for the home-care sector, our findings suggest that these tariffs should primarily be based on ADL and IADL impairments and mobility limitations – or on these impairments in combination with living alone – rather than on the presence of a dementia diagnosis or symptoms of cognitive impairment. However, these findings are not necessarily directly transferable into other health care systems, as our analyses only included the costs for home nursing care financed by the Swiss mandatory health insurance. This excludes other support services such as household help, meals on wheels, help with cooking or managing finances that are important in dementia patients and are financed in other countries. Our findings should therefore be reevaluated in other health care systems that include more assistance related tasks in their home care financing schemes.

Living alone was not a strong predictor of home nursing care costs in the univariate analyses. In all multivariate analyses, however - and particularly in the dementia subgroup - living alone emerged as an important predictor. It should therefore be considered as a predictor in tariff models, especially for people with dementia.

Results of supplementary analyses showed that the basic models could not markedly be improved by different categorisation of predictors or model specification (i.e. interactions). However, the basic model with additional predictors (including home care service provider identification, comorbidity and mobility) showed improved prediction and that home care service providers themselves were strong predictors of home care costs. These differences among providers might stem from a combination of reasons, including differences in population or unmeasured regional factors, organisational decisions or varying data collection standards. Such differences would have to be better understood and eliminated, if tariffs should be developed based on such real-world data.

### Strengths and limitations

Our dichotomous measure of cognitive impairment was rather crude. The measure just captured any observable sign of cognitive impairment above a CPS of 1, without differentiating for severity. This simplification was necessary due to the limited number of individuals with severe cognitive impairment in our sample, which is probably attributable to their higher likelihood of nursing home entries [21, 34–36]. However, the use of a more refined cognitive impairment categorisation in our supplementary analyses suggests that our initial conclusions hold. Still, a larger and more diverse dataset would allow for more nuanced analyses of how different levels of cognitive impairment affect home nursing care costs. It would also enable better examination of cognitive impairment among individuals without a formal dementia diagnosis, potentially uncovering predictive patterns currently obscured by sample limitations.

With regard to information on dementia diagnosis as well as all other diagnoses or symptoms we relied entirely on real-world data from the InterRAI. The data could be influenced by misclassifications, recording bias, incorrect entries, incompleteness of records or even effects of billing incentives, as this is typical in real-world studies [37, 38]. Concerning the dementia diagnosis, we used the symptoms-based CPS to counteract possible underdiagnosis that has been shown to be prevalent in dementia patients in home care [28, 29] or data problems at least partly. But this measure underlies the same data quality restrictions. Furthermore, by using the CPS we could have misclassified people that show cognitive impairment for other reasons than dementia. Data quality issues should, therefore, be scrutinised, addressed and improved in further analyses.

Even though home care nurses have very limited restrictions for their care planning in Switzerland, their care planning might still be influenced by the tariff system itself [38]. Thus, the level of costs in our data may be partly influenced by regulatory factors. For example. if nurses anticipated that a certain insurer would review their care planning if they planned more than 60 h of care in a quarter [26], they might have adjusted their care planning to remain below this threshold. In our data, we did not find any level effects around this threshold. However, we also did not have any information about the insurers and could not adjust for such bias. Therefore, we suggest that, in future research, such influences should be considered.

In our sample, individuals aged 80 years and older were overrepresented, while those under 65 years were underrepresented compared to the general Swiss home care population [33]. One likely explanation for the underrepresentation of the 20–64 age group is their generally better health status, which usually exempts them from completing the interRAI assessment. However, the prevalence of dementia in the younger age group is relatively low, and they typically exhibit fewer limitations in ADL and IADL. Therefore, this sampling bias is unlikely to substantially affect the validity of our findings. Nevertheless, including more individuals from the younger age group in future analyses could potentially strengthen our conclusions. Improving the generalisability of our results would require a broader dataset encompassing a more diverse patient population, ideally collected from all home care service providers across Switzerland.

## Conclusion

Our findings give no support for the implementation of differentiated reimbursement tariffs for dementia patients or patients with cognitive impairment. Other predictors of home nursing care costs such as ADL and IADL impairments or living alone seem to capture the everyday life hindrances dementia patients face adequately enough. Due to a small sample size especially in some subgroups, e.g. in dementia patients with strong cognitive impairment, further research with larger and more representative samples is necessary to validate and extend these findings. Furthermore, our findings are based on the financing scheme of the Swiss health care system and should therefore be reproduced in other countries. Finally, a more refined and detailed measurement of cognitive impairment, applied to a broader and more diverse population would enhance the generalisability and precision of such cost prediction models.

Overall, our study contributes to a better understanding of the economic implications of dementia in home care but also calls for further research in this field.

## Supplementary Information

Below is the link to the electronic supplementary material.

Supplementary Material 1

## Data Availability

The data analysed in this study is not publicly available due to data protection laws under which participating home care service providers operate (Art. 5c, digit 2, FADP 32).

## Abbreviations

ADL: Activities of daily living
CI: Confidence interval
CHF: Swiss Francs
IADL: Instrumental activities of daily living
interRAI: Interrai-Home Care Switzerland assessment questionnaire
OBMHI: Ordinance on Benefits in the Mandatory Health Insurance
SD: Standard deviation
