# Supplementary material for: Should we include dementia diagnosis or cognitive impairment to predict home care cost? An observational study using real-world data
Source: BMC Health Serv Res. 2025 Dec 22;26:112. doi: 10.1186/s12913-025-13868-2 (PMC12831370; doi:10.1186/s12913-025-13868-2)
Supplement: Supplementary file 1 — Supplementary Material 1 [file 12913_2025_13868_MOESM1_ESM.pdf]

Online supplement for:

Should we include dementia diagnosis or cognitive impairment to predict home care cost? An observational study using real-world data

Section A: Different categorisations of the cognitive performance scale (CPS) with a cut-off of CPS≥1 instead of CPS≥2 and with four categories and its changes to prediction models for home nursing care costs

Table A1

Description of cognitive performance scale (CPS) with four categories by dementia diagnosis status

	Dementia Diagnosis	
CPS with 4 categories	Yes	No
Intact	13	488
Borderline intact	29	203
Mild	80	138
Moderate or above	54	30
Total	176	859

Table A2:

Multivariable prediction of home care costs using a GLM models with a log-link and cognitive performance scale (CPS) with a cut-off of CPS 1 (any impairment)

	Basic Model				Model 1				Model 2				Model 3			
BIC	***-6281.88***				***-6280.97***				***-6283.03***				***-6278.91***			
AIC	***15.11***				***15.11***				***15.11***				***15.11***			
		CI				CI				CI				CI		
	coeff	lower	upper	p	coeff	lower	upper	p	coeff	lower	upper	p	coeff	lower	upper	p
Age	0.003	-0.002	0.008	0.239	0.003	-0.002	0.008	0.315	0.003	-0.002	0.008	0.280	0.003	-0.003	0.008	0.326
male gender	0.075	-0.043	0.192	0.215	0.090	-0.028	0.209	0.135	0.071	-0.047	0.189	0.236	0.083	-0.036	0.201	0.171
ADL impairment	0.143	0.119	0.167	<0.001	0.142	0.118	0.166	<0.001	0.142	0.118	0.166	<0.001	0.141	0.117	0.165	<0.001
IADL impairment	0.076	0.042	0.110	<0.001	0.069	0.034	0.103	<0.001	0.062	0.026	0.097	0.001	0.059	0.024	0.095	0.001
living alone	0.278	0.158	0.397	<0.001	0.273	0.153	0.392	<0.001	0.267	0.147	0.386	<0.001	0.265	0.146	0.385	<0.001
dementia diagnosis					0.206	0.053	0.359	0.009					0.149	-0.011	0.309	0.068
cognitive impairment									0.187	0.070	0.304	0.002	0.151	0.029	0.274	0.016

ADL: activities of daily living, AIC: Akaike Information Criterion, BIC: Bayesian Information Criterion, CI: Confidence Interval, IADL: instrumental activities of daily living

Table A3:

Multivariable prediction of home care costs using a GLM models with a log-link and cognitive performance scale (CPS) with four categories

	Basic Model				Model 2 (CPS with 4 categories)				Model 3 (CPS with 4 categories)			
BIC	***-6281.88***				***-6269.62***				***-6265.21***			
AIC	***15.11***				***15.11***				***15.11***			
		CI				CI				CI		
	coeff	lower	upper	p	coeff	lower	upper	p	coeff	lower	upper	p
Age	0.003	-0.002	0.008	0.239	0.003	-0.002	0.008	0.297	0.002	-0.003	0.008	0.333
male gender	0.075	-0.043	0.192	0.215	0.074	-0.044	0.192	0.218	0.084	-0.034	0.203	0.164
ADL impairment	0.143	0.119	0.167	<0.001	0.142	0.117	0.166	<0.001	0.142	0.117	0.167	<0.001
IADL impairment	0.076	0.042	0.110	<0.001	0.061	0.025	0.097	0.001	0.060	0.024	0.095	0.001
living alone	0.278	0.158	0.397	<0.001	0.268	0.148	0.388	<0.001	0.264	0.144	0.384	<0.001
dementia diagnosis									0.152	-0.020	0.325	0.084
cognitive impairment												
Borderline intact					0.155	0.010	0.299	0.036	0.147	0.002	0.292	0.047
Mild					0.220	0.004	0.070	0.369	0.166	0.006	0.327	0.043
Moderate or above					0.195	0.095	-0.034	0.423	0.109	-0.138	0.356	0.386

ADL: activities of daily living, AIC: Akaike Information Criterion, BIC: Bayesian Information Criterion, CI: Confidence Interval, IADL: instrumental activities of daily living

Section B: Predictive models with a quadratic term for age added

Table B1

Multivariable prediction of home care costs using a GLM models with a log-link and with a quadratic term for age

	**Basic Model (including age²)**				**Model 2 (including age²)**				**Model 3 (including age²)**				**Model 4 (including age²)**			
BIC	***-6274.98***				***-6274.04***				***-6272.80***				***-6268.59***			
AIC	***15.11***				***15.11***				***15.11***				***15.11***			
		CI				CI				CI				CI		
	coeff	Lower	upper	p	coeff	lower	upper	p	coeff	lower	upper	p	coeff	lower	upper	p
age	0.007	-0.028	0.042	0.698	0.004	-0.031	0.040	0.811	0.004	-0.031	0.040	0.814	0.003	-0.032	0.039	0.856
age^2	0.000	0.000	0.000	0.824	0.000	0.000	0.000	0.921	0.000	0.000	0.000	0.929	0.000	0.000	0.000	0.963
male gender	0.075	-0.043	0.192	0.215	0.090	-0.028	0.209	0.135	0.080	-0.037	0.198	0.181	0.090	-0.029	0.208	0.138
ADL impairment	0.144	0.119	0.168	<0.001	0.142	0.118	0.166	<0.001	0.141	0.117	0.165	<0.001	0.141	0.116	0.165	<0.001
IADL impairment	0.076	0.042	0.111	<0.001	0.069	0.034	0.103	<0.001	0.068	0.032	0.103	<0.001	0.065	0.030	0.100	<0.001
living alone	0.279	0.159	0.398	<0.001	0.273	0.153	0.393	<0.001	0.279	0.160	0.399	<0.001	0.275	0.155	0.395	<0.001
dementia diagnosis					0.206	0.052	0.359	0.009					0.155	-0.015	0.326	0.073
cognitive impairment									0.155	0.027	0.283	0.018	0.096	-0.046	0.239	0.185

ADL: activities of daily living, AIC: Akaike Information Criterion, BIC: Bayesian Information Criterion, CI: Confidence Interval, IADL: instrumental activities of daily living

Section C: Predictive models with an interaction term between ADL impairment and dementia diagnosis and/or cognitive impairment

Table C1

Multivariable prediction of home care costs using a GLM models with a log-link and an interaction between ADL impairment and dementia diagnosis and/or cognitive impairment (only Basic Model and Model 2)

	**Basic Model**				**Model 2 (including interaction ADL impairment*dementia diagnosis)**			
BIC	***-6281.88***				***-6278.77***			
AIC	***15.11***				***15.11***			
		CI				CI		
	coeff	lower	upper	p	coeff	lower	upper	p
age	0.003	-0.002	0.008	0.239	0.003	-0.002	0.008	0.306
male gender	0.075	-0.043	0.192	0.215	0.089	-0.029	0.208	0.140
ADL impairment	0.143	0.119	0.167	<0.001	0.156	0.129	0.183	<0.001
IADL impairment	0.076	0.042	0.110	<0.001	0.070	0.035	0.105	<0.001
living alone	0.278	0.158	0.397	<0.001	0.262	0.142	0.382	<0.001
dementia diagnosis					0.387	0.169	0.606	0.001
ADL impairment*dementia diagnosis					-0.062	-0.112	-0.012	0.016
cognitive impairment								
ADL impairment*cognitive impairment								

ADL: activities of daily living, AIC: Akaike Information Criterion, BIC: Bayesian Information Criterion, CI: Confidence Interval, IADL: instrumental activities of daily living

Section D: Predictive models with an interaction term between gender and dementia diagnosis and/or cognitive impairment

Table D1

Multivariable prediction of home care costs using a GLM models with a log-link and an interaction between gender and dementia diagnosis and/or cognitive impairment

	**Basic Model**				**Model 2 (including interaction gender*dementia diagnosis)**				**Model 3 (including interaction gender*cognitive impairment)**				**Model 4 (including including interaction gender*dementia diagnosis and gender*cognitive impairment)**			
BIC	***-6281.88***				***-6275.15***				***-6275.83***				***-6264.06***			
AIC	***15.11***				***15.11***				***15.11***				***15.11***			
		CI				CI				CI				CI		
	coeff	lower	upper	p	coeff	lower	upper	p	coeff	Lower	upper	p	coeff	lower	upper	p
age	0.003	-0.002	0.008	0.239	0.003	-0.002	0.008	0.316	0.003	-0.002	0.008	0.281	0.003	-0.002	0.008	0.316
male gender	0.075	-0.043	0.192	0.215	0.118	-0.010	0.245	0.070	0.151	0.012	0.290	0.033	0.154	0.014	0.294	0.031
ADL impairment	0.143	0.119	0.167	<0.001	0.142	0.118	0.166	<0.001	0.141	0.117	0.166	<0.001	0.141	0.117	0.165	<0.001
IADL impairment	0.076	0.042	0.110	<0.001	0.069	0.034	0.103	<0.001	0.069	0.034	0.104	<0.001	0.067	0.032	0.102	<0.001
living alone	0.278	0.158	0.397	<0.001	0.269	0.149	0.389	<0.001	0.278	0.159	0.159	<0.001	0.273	0.153	0.392	<0.001
dementia diagnosis					0.262	0.080	0.445	0.005					0.160	-0.052	0.373	0.140
male gender*dementia diagnosis					-0.192	-0.516	0.131	0.244					-0.078	-0.434	0.279	0.669
cognitive impairment									0.245	0.086	0.403	0.002	0.170	-0.015	0.356	0.072
male gender*cognitive impairment									-0.247	-0.499	0.005	0.054	-0.191	-0.472	0.090	0.184

ADL: activities of daily living, AIC: Akaike Information Criterion, BIC: Bayesian Information Criterion, CI: Confidence Interval, CPS: Cognitive Performance Scale, IADL: instrumental activities of daily living
